# Effect of High Hydrostatic Pressure Treatment on Urease Activity and Inhibition of Fishy Smell in Mackerel (*Scomber japonicus*) during Storage

**DOI:** 10.4014/jmb.2106.06052

**Published:** 2021-09-18

**Authors:** Han-Ho Kim, Si-Hyeong Ryu, So-Mi Jeong, Woo-Sin Kang, Ji-Eun Lee, Su-Ryong Kim, Xiaotong XU, Ga-Hye Lee, Dong-Hyun Ahn

**Affiliations:** 1 Department of Food Science and Technology and Institute of Food Science, Pukyong National University, Busan 48513, Republic of Korea; 2 Institute of Fisheries Sciences, Pukyong National University, Busan 46041, Republic of Korea

**Keywords:** High hydrostatic pressure, urease, *Vibrio parahaemolyticus*, fishy smell, mackerel

## Abstract

In this study, the physicochemical changes related to fishy smell were determined by storing high hydrostatic pressure (HHP)-treated mackerel (*Scomber japonicus*) meat in a refrigerator for 20 days. The inhibition of crude urease activity from *Vibrio parahaemolyticus* using HHP treatment was also investigated. The mackerel meat storage experiment demonstrated that production of trimethylamine (TMA) and volatile basic nitrogen (VBN), the main components of fishy smell, was significantly reduced on the 20th day of storage after the HHP treatment compared to the untreated mackerels. The results demonstrated that the increased ammonia nitrogen rates in the 2000, 3000, and 4000 bar, HHP-treated groups decreased by 23.8%, 23.8%, and 31.0%, respectively, compared to the untreated groups. The enzyme activity of crude urease was significantly reduced in the HHP-treated group compared to that in the untreated group. Measurement of the volatile organic compounds (VOCs) in mackerel meat during storage indicated that the content of ethanol, 2-butanone, 3-methylbutanal, and trans-2-pentenal, which are known to cause off-flavor due to spoilage, were significantly reduced by HHP treatment. Collectively, our results suggested that HHP treatment would be useful for inhibiting the activity of urease, thereby reducing the fishy smells from fish and shellfish.

## Introduction

Fishes start losing freshness after death, and the content of TMA, ammonia, and VBN, which are the substances that cause fishy odor because of the activation of microorganisms, gradually increases. Thus, in the absence of appropriate processing mechanisms, the time by activating for consumption, is limited [1].

High hydrostatic pressure (HHP), from the isostatic pressing process long used for ceramic materials, is now being applied to the food industry. Variations of the HHP process depend on the pressure-transmitting fluid (water or gas), temperature, and the specific product. The applied pressure and treatment time will depend on the type of product being processed and the expected final product. In general, for enzyme inactivation, a pressure higher than the pressure used for microorganism inactivation should be used [2].

HHP treatment technology to improve food storage and preference is one of the technologies that can provide safer, high-quality, and value-added food than other non-thermal treatment technologies (*e.g.*, UV irradiation, low-temperature osmotic dehydration, radiation, etc.) [3].

Fish spoilage occurs because of the presence of endogenous enzymes and spoilage microorganisms. It leads to rapid quality deterioration of fresh fish during handling and storage and limited shelf life of the product [4]. HHP treatment inhibits the activity of microorganisms by breaking non-covalent bonds and changing the permeability of cell membranes, resulting in enzyme inactivation. The effects on non-covalent binding, along with changes in cell membrane permeability, result in microbial reduction, enabling safe products with extended shelf life without affecting the nutritional properties and flavor [5-7].

Consequently, the application of HHP treatment technology has been increasing recently, and various effects of HHP treatment on fish have been reported depending on different factors, such as the nature of the species and its size and chemical composition [8, 9].

Products studied by introducing HHP treatment technology include fruits, salad dressings, yogurt, juice, and processed products using rice [10]. In addition, other studies based on HHP treatment of fisheries products have reported enhancement of storage properties of seasoned squid [11], inhibition of microbial growth in raw oysters [12], changes in the quality and microbiological changes of kochujang-gulbi (dried croaker with red pepper paste)[13], and inhibition of microbial growth in mackerel (*Scomber japonicus*) [14].

Mackerel, along with sardine (*Sardinops sagax*), Jack mackerel (*Trachurus japonicus*), and Pacific saury (*Cololabis saira*), are four fishes with external blue coloring. Mackerel is rich in lipids and proteins and contains a large number of nucleic acids, which facilitate cell division and replication, and large amounts of taurine, which is known to be effective in preventing stroke. In particular, the content of polyunsaturated fatty acids, such as EPA and DHA, is very high in mackerel [15-17]. Mackerel is considered to have high nutritional value due to its high polyunsaturated fatty acid content, which promotes brain activity and has beneficial effects in several diseases, such as arteriosclerosis and myocardial infarction [18].

However, it is known that mackerel has a very high content of unsaturated fatty acids, which causes quick development of rancidity, protein denaturation, denaturation of lipids, and free fatty acid formation, resulting in occurrence of fishy smell faster than in other fish species. It has also been reported that histamine produced by microorganisms in mackerel causes scombroid fish poisoning, a type of food poisoning [19, 20].

On the other hand, high levels of urea are found in the muscles of elasmobranch fishes such as rays and sharks, which is hydrolyzed by the urease secreted by microorganisms to produce ammonia and carbon dioxide [21]. Owing to the reaction of the urease enzyme, the pH of the fish meat increases and becomes alkaline and the volatility increases, resulting in a strong ammonia-like smell [22, 23]. Therefore, to control the foul smell of fish and shellfish, it is important to reduce the microorganisms that play a key role in the occurrence of fishy smell, and to inhibit the activity of related enzymes and oxidation. However, among the enzymes that cause fishy smell (lipase and lipoxygenase), studies on urease are relatively insufficient.

In this study, the effect of HHP treatment on fishy smell inhibition in mackerel meat was investigated during a storage period of 20 days. Using HHP treatment, we also attempted to verify the possibility of inhibiting the activity of urease, known as a fishy smell-related enzyme, and to control substances that cause fishy smell of mackerel during refrigerated storage.

## Materials and Methods

### Mackerel Samples

Mackerel (*Scomber japonicus*) was purchased from a local market (Korea). The skin, gill, and viscera of the mackerel were aseptically removed on a clean bench. The meat was rinsed with sterile distilled water, placed in a sterile blender and ground with 0.05% ascorbic acid at 1,000 rpm for 30 s in an ice box.

### Preparation for Crude Urease Enzyme Extraction

Crude urease enzyme was extracted from *Vibrio parahaemolyticus* according to the method described by Kim *et al*. [24]. *V. parahaemolyticus* was isolated and identified from *Saccharina japonica* collected from Songjeong, Busan. To verify the urease production in *V. parahaemolyticus*, Christensen’s urea agar and Bacto urea broth (Difco, USA) were used and cultured in lysogeny broth (LB) (pH 5.5) supplemented with 2% NaCl, 0.5% glucose, and 0.2% urea. After adding 2–3% of the pre-culture solution to the LB medium, it was incubated at 37°C for 6–7 h, followed by centrifugation at 6,992 ×*g* for 10 min at 4°C. The precipitate was washed twice with saline solution and washed with 20 mM phosphate buffer (pH 7.0). The cells were crushed using an ultrasonic crusher, centrifuged at 15,729 ×*g* for 10 min at 4°C, and the supernatant was used as crude urease enzyme.

### HHP Treatment

The mackerel meat and crude urease enzyme were vacuum-packed and placed on ice to minimize any temperature effects prior to HHP treatment. The samples were processed in a pressure chamber (QFP 215 L-600 High Pressure Processing System, AVURE Technologies Inc., USA) with a volume of 215 L, and with tap water as the pressure transfer fluid. The pressure time was approximately 18-20 s per 1000 bar, and the pressure release time was less than 35 s. The initial water temperature was 19°C, and the increase in temperature due to adiabatic heating was 2°C per 1000 bar. The treatment groups were high-pressure treated in batches at 2000, 3000, and 4000 bar for 3 min, but the control group was not treated. Compression and decompression times were not included in the treatment. After treatment, the samples were stored at 4°C for 20 days, and TMA, VBN, pH, color values, volatile organic compounds (VOCs), and ammonia nitrogen were measured at 10-day intervals.

### Measurement of Urease Enzyme Activity

Urease enzyme activity was measured using the method described by Weatherburn [25]. Briefly, decomposition of urea as a substrate results in production of ammonia, which reacts with phenol in the presence of hypochlorite to form indophenol that is quantified colorimetrically. Fifty microliters of crude urease enzyme and 200 μl of UHEP (20 mM HEPES buffer, pH 7.5, containing 30 mM urea, 1 mM EDTA, and 1 mM 2-mecaptoethanol) were mixed and incubated at 37°C for 30 min. Subsequently, 400 μl of phenol nitroprusside and 400 μl of alkaline hypochlorite were added to the reaction mixture and reacted at 50°C for 10 min, followed by measurement of absorbance at 625 nm. Urease activity was calculated by comparing the absorbance value with the standard curve for each unit of Jack bean (*Canavalia ensiformis*) urease (U1500, Sigma-Aldrich Chemical Co., USA).

### Measurement of TMA

The TMA content of HHP-treated mackerel meat was measured by modifying the AOAC (Association of Official Analytical Chemists) method [26]. HHP-treated (10 g) or untreated mackerel meat was added to 20 ml of 7.5% TCA (trichloroacetic acid) solution and then homogenized using a homogenizer (AM-7, Ace Homogenizer, Japan) for 1 min at 5,000 rpm. Each sample was stirred gently, centrifuged at 1,977 ×*g* for 10 min, and then filtered. The filtrate (4 ml) was mixed with 1 ml of 20% formaldehyde, 10 ml of anhydrous toluene, and 3 ml of saturated K_2_CO_3_ solution followed by vortexing for 1 min. The mixed sample was allowed to stand for 5 min, and the separated toluene supernatant was dehydrated by adding Na_2_SO_4_ for 1 min. The dehydrated supernatant was reacted with 0.02% picric acid (1:1 ratio), and the absorbance was measured at 410 nm.

### Measurement of VBN

The VBN content was measured by modifying Conway's method of the Korean Food Standards Codex [27]. HHP-treated (10 g) or untreated mackerel meat was added to 50 ml of distilled water and then extracted for 30 min. The mixture was filtered and made up to 100 ml with distilled water. Diffusion and titration reactions were carried out, wherein 1 ml of test liquid was poured into the bottom of the left side of the outer well and 1 ml of 0.01 N H_2_SO_4_ was added into the inner well of the Conway unit. Vaseline gel was applied at the contact part of the cover plate and the outer well. The cover plate was closed to approximately 2/3, and 1 ml of saturated K_2_CO_3_ solution was added to the right side of the outer well. The cover plate was then rapidly closed and fixed by a clip. The Conway unit was shaken to mix the outer well solution equally and incubated at 25°C for 1 h. The cover plate was opened, and one drop of the Brunswik indicator was added to the inner well. This was titrated by 0.01 N NaOH using a micro burette until the color changed to green. The titration was repeated twice.

### Measurement of Ammonia Nitrogen

The ammonia nitrogen content was measured according to the method described by Kim *et al*. [24]. HHP-treated (10 g) or untreated mackerel meat was added to 100 ml of hot water, stirred for 10 min, and heated in a water bath (100°C) for 1 min. Then, distilled water was added to a final volume of 200 ml, mixed, and filtered through a filter paper (Advantec No. 2, Japan), and the filtrate was used as a sample solution. To measure the ammonia nitrogen, 0.1 ml of the sample solution and 2 ml of each of the solutions A (containing 1% phenol and 0.005% sodium nitroprusside dehydrate in distilled water), and B (containing 0.9% disodium hydrogen phosphate, 0.6% sodium hydroxide, and 1% sodium hypochlorite in distilled water), were mixed and reacted at 37°C for 20 min, and the absorbance was measured at 630 nm.

### Measurement of Color Values and pH

The color values of mackerel meat treated with HHP were measured as lightness (L*), yellowness (b*), and redness (a*) using a colorimeter (JC 801, Color Techno System Co., Japan). The measurement was repeated at least five times to obtain an average value. The values of the standard color plate used were L * = 98.98, a * = 0.21, and b * = -0.28.

HHP-treated (3 g) or untreated mackerel meat was added to 30 ml of distilled water and homogenized with a homogenizer for 2 min at 10,000 rpm. The pH of the homogenized samples was measured at room temperature using a pH meter (*n* = 5) (HM-25V, TOA, Japan).

### Analysis of VOCs

The VOCs of the HHP-treated samples were analyzed under the conditions listed in Table 1 using an automatic thermal desorber (ATD650, Perkin Elmer, USA) and a gas chromatography-mass spectrometer (TQ8050, Shimadzu, Japan). The identities of the volatile compounds were matched with literature data (Willey/NBS Registry of Mass Spectra Data and Eight Peak Index of Mass Spectra) and the GCQ library search system (National Institute of Standards and Technology (NIST) mass spectra database). Compounds showing < 80% similarities and low peak values were classified as unknown substances.

### Statistical Analyses

Data were expressed as the mean ± SEM (*n* = 3). Statistical evaluation was carried out using analysis of variance with SAS software (ver. 9.4, SAS Institute, Inc., USA), according to Duncan's multiple range test (*p* < 0.05).

## Results and Discussion

### Effect of HHP Treatment on TMA Levels

Trimethylamine oxide (TMAO) in fish and shellfish is reduced to TMA by bacteria or enzymes after death, causing a fishy smell, and through this, the degree of spoilage is measured [28, 29]. TMA has a higher rate of increase in its production than ammonia, so it is a good indicator of freshness. In general, if the TMA content is more than 3-4 mg/100 g, it is judged to be initial spoilage, but the limit of initial spoilage varies greatly depending on the fish species, so in the case of Pacific herring, up to 7 mg/100 g is allowed [30].

In this study, the changes in TMA levels during storage at 4°C in HHP-treated or untreated mackerel meat are reported in Table 2. On day 0, no significant difference was observed between the treatment and untreated groups, but on days 10 and 20 of storage, the TMA content significantly decreased as the treatment pressure increased. On the 10th day of storage, the TMA content decreased by 12.90%, 71.67%, and 87.24% when treated with 2000, 3000, and 4000 bar, respectively, compared to the untreated group. In addition, on the 20th day of storage, it was observed that the TMA content decreased by 12.21%, 35.8%, and 70.27%, when treated with 2000, 3000, and 4000 bar, respectively, compared to the untreated group. In particular, freshness was maintained during storage for 20 days in the case of the 4000-bar treatment.

These results were similar to those of Gou *et al*. [11], wherein the TMA content in seasoned squid subjected to HHP treatment was decreased by 77%, 71%, and 69% compared to the untreated group on days 7, 10, and 21 of storage, respectively. Several studies have reported that TMA content increases in proportion to the growth of microorganisms and decreases the quality of fish [28, 29]. Therefore, these results suggest that microbial growth is inhibited by HHP treatment, thereby inhibiting the production of TMA.

### Effect of HHP Treatment on VBN Levels

When fresh mackerel is spoiled, the quantity of VBN contained in trace amounts in the meat increases. As fish decay progresses, proteins are decomposed into low-molecular substances, such as peptides, amino acids, and peptones, and the content of VBN increases [31]. In addition, as TMAO is reduced to basic substances, such as TMA, by enzymes and microorganisms, the content of VBN increases [32].

In this study, HHP-treated or untreated mackerel samples were stored at 4°C for 20 days, and the changes in VBN content were measured according to storage days (Table 3). On day 0, no significant differences were observed between the treated and untreated groups. However, on the 10th and 20th days of storage, it was observed that the VBN content significantly decreased as the treatment pressure increased. In the case of the untreated group, the VBN content was 27.40 mg/100 g on the 10th day of storage, which was an initial level of spoilage, and 53.79 mg/100 g on the 20th day of storage, which was found to be the level of complete spoilage. By contrast, when treated with pressure of 2000, 3000, and 4000 bar, the VBN contents on the 10th day of storage were 21.84, 19.56, and 18.93 mg/100 g, respectively. Compared to the control group, the rates of the treated group were decreased by 20.29%, 23.61%, and 30.91%, respectively. Even on the 20th day of storage, in the case of the group treated with 4000 bar pressure, the VBN content was 26.07 mg/100 g, maintaining the normal level of freshness.

In general, when the VBN content is more than 50 mg/100 g, it is considered complete spoilage [33]. The VBN content at the level of complete spoilage was demonstrated on the 20th day in the untreated group. As the treatment pressure increased, the rate of increase in VBN content significantly decreased compared to the control group. The VBN content of the group treated with 4000 bar pressure decreased by approximately 51.53%compared to the untreated group. These results are similar to those of Kang *et al*. [13], who treated kochujang-gulbi with HHP and showed that the rate of increase in the VBN content of HHP-treated kochujang-gulbi samples (at 6000 bar, 20°C for 10 min) decreased by approximately 21.25% compared to that in the untreated group.

### Effect of HHP Treatment on Color Values

The color change of mackerel meat by HHP is shown in Table 4. The lightness value (L*) of the untreated group increased significantly from 34.16 on day 0 to 37.70 on day 20 as the storage period increased. In contrast, the initial L* values of the 2000, 3000, and 4000 bar treatment groups were 37.66, 44.21, and 45.67, respectively, which were significantly higher than those of the untreated group. On the 20th day of storage, the L* values of the 3000 and 4000 bar treatment groups were 42.86 and 44.82, respectively, which were significantly decreased compared to those observed on day 0. On day 0, a* and b* values tended to be slightly decreased by the HHP treatment, but no significant change was observed during the storage period according to the pressure treatment.

This result is similar to the study by Cruz-Romero *et al*. [34] which showed that the lightness increases after ultra-hydrostatic treatment of 1000-8000 bar on oysters. In addition, Kang *et al*. [32] reported that the lightness of mackerel samples increased with storage time by combined treatment with *Citrus junos* or *Prunus mume* immersion solution and HHP in mackerel. Choi *et al*. [35] also reported that the lightness of meat products increased due to the irreversible denaturation of proteins when treated with HHP above 3000 bar. Furthermore, it has been reported that the denaturation of myofibrillar and sarcoplasmic proteins is related to the change in lightness [36]. Therefore, we report in this study that the increase in lightness by HHP treatment is due to the degeneration of mackerel muscle protein.

### Effect of HHP Treatment on pH Values

The pH value of fresh fish after death is usually around 5.5-6.5 [37], and the pH increases over time. The pH value increases because various enzymes in fish degrade meat protein or the protein in fish is decomposed by contaminated microorganisms, resulting in an increase in amino, ammonia nitrogen, peptides, amino acids, and amines [38].

In this study, we found that the pH values of the untreated group and the 2000 bar-treated group significantly increased from 5.49 to 6.47 on day 0, and 5.59 to 6.25 on day 20 (Table 5). However, the untreated group and the 2000 bar-treated group showed initial spoilage pH values on day 20 of storage. The usual pH range for the initial spoilage of red meat fish is between 6.2–6.4, and any value beyond 6.5 is considered inconsumable [30]. In contrast, though the pH values of the 3000 bar and 4000 bar-treated groups also increased from 5.66 to 5.86 and 5.68 to 5.99, respectively, they remained below the 6.2 initial spoilage mark.

These results were similar to those reported by Reyes *et al*. [39], which showed the change in pH value in untreated and HHP-treated Chilean jack mackerel samples during chilled storage for 26 days. The authors explained that the lower pH value found in pressurized samples compared to untreated samples might be attributable to the total inactivity of *Shewanella putrefaciens*, which is considered to be the major TMA-producing bacteria in seafood. It is widely known that an increase in the pH value of fish muscle leads to the accumulation of undesirable alkaline compounds, such as ammonia and TMA, which are primarily derived from microbial activity [40].

### Effect of HHP Treatment on Ammonia Nitrogen Levels

As a result of measuring the ammonia nitrogen content during the storage period of 0–20 days (Table 6), the ammonia nitrogen content in both the untreated group and all HHP-treated groups was significantly increased. However, it was established that the increased rates of ammonia nitrogen in the 2000, 3000, and 4000 bar HHP-treated groups decreased by 23.8%, 23.8%, and 31.0%, respectively, compared to the untreated groups. In addition, on days 0 and 10, the content of ammonia nitrogen was significantly decreased in samples treated with 4000 bar pressure compared to that in the untreated group, and on the 20th day, the ammonia nitrogen content of all HHP-treated groups significantly decreased compared to the untreated group.

In aquatic organisms, various products are produced as final metabolites of nitrogen compounds, like ammonia. In general, when the freshness of fish decreases, a severe ammonia odor occurs because the urea contained in muscles is decomposed by urease secreted by bacteria to produce ammonia [41]. When foods are exposed to extremely high pressures, microbes are removed in the same way as heat treatment. HHP treatment inactivates bacterial cells by interfering with the basic cellular functions essential for microbial reproduction and survival. HHP treatment can disrupt microbial cell membranes, hampering the transport of nutrients and waste products. If important enzymes are inhibited or if the selective permeability of the membrane is decreased, vital cellular processes are altered [42].

Therefore, the urease activity secreted by microorganisms present in fish can be inhibited by high hydrostatic treatment, thereby suppressing the fishy smell.

### Urease Inhibition Activity

The activity of the crude urease enzyme extracted from *V. parahaemolyticus* treated with HHP is shown in Table 7. The crude urease enzyme activity of the untreated group and the 2000, 3000, and 4000 bar HHP treatment groups were 25.40, 21.36, 19.79, and 20.88 unit/mg, respectively. This result indicated that urease enzyme activity was significantly reduced in the HHP-treated than in the untreated group. However, there was no significant difference in the activity based on the treatment pressure. Usually, the urea contained in fish is decomposed by the urease secreted by microorganisms to produce ammonia. A fishy smell is generated by increase in the pH of fish meat by ammonia and increasing volatility [22, 23].

The results of this study demonstrated that the urease activity was inhibited by HHP treatment, and it can be assumed that HHP treatment will be useful for the inhibition of fishy smell by inhibiting urease activity.

### Effect of HHP Treatment on VOCs

To evaluate the change in the contents of VOCs in HHP-treated mackerel meat during storage for 20 days, a total of 38 VOCs were detected, including 14 aldehydes, 5 alcohols, 4 ketones and 15 other compounds (data not shown). The contents of ethanol, 2-butanone, 3-methylbutanal, and trans-2-pentenal, which are known to cause off-flavor due to fish spoilage, are shown in Table 8. On day 0, the ethanol content in the groups treated with 2000 and 3000 bar pressure decreased by 37.93% and 8.9%, respectively, compared to the untreated group, whereas it increased by 3.54% in the group treated with 4000 bar pressure compared to the untreated group. During storage for 20 days, the ethanol content of the untreated group and the groups treated with 2000, 3000, and 4000 bar pressure increased by 679.28%, 86.96%, 52.65%, and 26%, respectively. These results suggested that the increase in ethanol content by HHP treatment at 2000, 3000, and 4000 bar decreased by 87.20%, 92.24%, and 96.08%, respectively, compared to the untreated group.

Ethanol is a common volatile compound generated by the microbial decay of fish [43-45]. Lerke and Huck [46] studied the quality of canned tuna using gas chromatography analysis and reported that the ethanol content increased with the spoilage of fish.

During storage for 20 days, the contents of 2-butanone in the untreated group and the groups treated at 2000, 3000, and 4000 bar increased by 209.18%, 128.51%, 69.19%, and 109.28%, respectively. The results indicated that the increase in the rate of 2-butanone content by 2000, 3000, and 4000 bar HHP treatment was decreased by 38.56%, 66.92%, and 47.75%, respectively, compared to the untreated group. Subsequently, Jonsdottir *et al*.[47] reported that 2-butanone had the highest concentration in the samples with the highest spoilage levels and was associated with off-flavor properties.

3-methylbutanal content was detected in any group on day 0. The content of 3-methylbutanal in the groups treated with 2000, 3000, and 4000 bar pressure decreased by 89.33%, 84.46%, and 88.0 3%, respectively, compared to the untreated group on the 10th day of storage and by 88.34%, 89.82%, and 91.58%, respectively on the 20th day of storage.

Previous studies have reported that the production of various short-chain alcohols, aldehydes, and ketones, such as ethanol, 3-methylbutanal, and 2-butanone, is a result of metabolic activity during fish spoilage by microorganisms [47-50]. In addition, these compounds have previously been suggested as indicators of microbial spoilage in smoked salmon [49, 50].

Therefore, HHP treatment is believed to delay the spoilage of fish by microorganisms, thereby reducing off-flavor, and our study suggested the possibility of inhibiting the action of urease by reducing the microorganisms.

## Figures and Tables

**Table 1:** Operating conditions of GC-MS (gas chromatography-mass spectrometry).

GC/MS (QP- 2010, Shimadzu, Japan)	
Oven temp	35℃ (10 min) 8℃/ min - 120℃ (10 min) 12℃/ min - 180℃ (7 min) 15℃/ min - 230℃ (10 min)
Column	At-1 60 m X 0.32 mm X 1.0 μm
Ion Source Temp	200℃
Interface Temp	250℃
Solvent Cut Time	1.00 min
Detector Gain Mode	Relative to the Tuning Result
Detector Gain	+0.00 kV
Threshold	0

**Table 2:** Changes in trimethylamine (TMA) of mackerel treated with high hydrostatic pressure (HHP) during storage at 4°C. (mg/ 100 g)

Storage period (days)	Treatments (bar)			

	0	2000	3000	4000
0	1.327±0.070^Ba^	1.297±0.020^Ba^	1.314±0.045^Ca^	1.323±0.174^Ba^
10	8.928±0.045^Aa^	7.776±0.038^Ab^	2.529±0.335^Bc^	1.139±0.043^Bd^
20	9.036±0.023^Aa^	7.932±0.108^Ab^	5.839±0.030^Ac^	2.686±0.206^Ad^

Means in the same column (A-C) and row (a-b) with different superscript letters are significantly different (*p* < 0.05).

**Table 3:** Changes in volatile basic nitrogen (VBN) levels of mackerel treated with high hydrostatic pressure (HHP) during storage at 4°C. (mg/ 100 g)

Storage period (days)	Treatments (bar)			

	0	2000	3000	4000
0	12.67 ± 5.44^Ac^	10.99 ± 3.46^Ac^	12.39 ± 1.88^Ac^	13.3 ± 1.08^Ac^
10	27.40 ± 1.03^Ab^	21.84 ± 0.98^Bb^	19.56 ± 0.24^BCb^	18.93 ± 0.84^Cb^
20	53.79 ± 1.33^Aa^	39.69 ± 1.68^Ba^	31.85 ± 2.07^Ca^	26.07 ± 1.83^Da^

Means in the same column (a-c) and row (A-D) with different superscript letters are significantly different (*p* < 0.05).

**Table 4:** Changes in color value of mackerel treated with high hydrostatic pressure (HHP) during storage at 4°C.

Color value	Treatments (bar)	Storage period (days)		

		0	10	20
L*	0	34.16±0.83^Dc^	35.66±0.57^Db^	37.70±0.39^Da^
	2000	37.66±0.31^Cb^	36.96±0.24^Cc^	38.86±0.26^Ca^
	3000	44.21±0.46^Ba^	43.25±0.21^Bb^	42.86±0.23^Bc^
	4000	45.67±0.74^Aa^	46.03±0.23^Aa^	44.82±0.41^Ab^
a*	0	11.80±0.23^Aa^	11.98±0.85^Aa^	11.08±0.22^Ab^
	2000	11.28±0.29^Ba^	10.19±1.66^Bb^	10.42±0.87^Bab^
	3000	10.59±0.21^Cb^	11.61±0.19^Aa^	10.69±0.38^ABb^
	4000	10.09±0.49^Da^	7.96±0.28^Cc^	9.32±0.75^Cb^
b*	0	9.46±0.27^Ac^	9.86±0.15^Db^	11.10±0.37^Ba^
	2000	8.39±0.35^Cb^	10.91±0.45^Ba^	11.12±0.14^Ba^
	3000	8.72±0.11^Bc^	10.29±0.23^Cb^	10.79±0.66^Ba^
	4000	7.77±0.31^Db^	12.93±0.60^Aa^	12.69±0.42^Aa^

Means in the same column (A-D) and row (a-c) with different superscript letters are significantly different (*p* < 0.05).

**Table 5:** Changes in pH of mackerel treated with high hydrostatic pressure (HHP) during storage at 4°C.

Storage period (days)	Treatments (bar)			

	0	2000 bar	3000 bar	4000 bar
0	5.49±0.01^Cc^	5.59±0.00^Bc^	5.66±0.03^Ac^	5.68±0.01^Ab^
10	6.18±0.02^Ab^	5.85±0.02^Bb^	5.78±0.02^Cb^	5.76±0.06^Cb^
20	6.47±0.04^Aa^	6.25±0.03^Ba^	5.86±0.09^Ca^	5.99±0.31^Ba^

Means in the same column (a-c) and row (A-C) with different superscript letters are significantly different (*p* < 0.05).

**Table 6:** Changes in ammonia nitrogen content of mackerel treated with high hydrostatic pressure (HHP) during storage at 4°C. (mg/ 100 g)

Storage period (days)	Treatments (bar)			

	0	2000 bar	3000 bar	4000 bar
0	0.11±0.04^Ac^	0.10±0.02^Ac^	0.08±0.02^ABc^	0.06±0.01^Bc^
10	0.28±0.01^Ab^	0.26±0.01^Ab^	0.26±0.01^Ab^	0.24±0.01^Bb^
20	0.53±0.01^Aa^	0.42±0.02^Ba^	0.40±0.05^Ba^	0.35±0.04^Ca^

Means in the same column (a-c) and row (A-C) with different superscript letters are significantly different (*p* < 0.05).

**Table 7:** Urease enzyme activity according to high hydrostatic pressure (HHP) treatment. (Unit/ mg)

	Treatments (bar)			

	0	2000	3000	4000
Urease enzyme activity	25.40±0.59^A^	21.36±2.03^B^	19.79±2.48^B^	20.88±1.06^B^

Means in the same column (A-B) with different superscript letters are significantly different (*p* < 0.05).

**Table 8:** Changes in volatile organic compounds (VOCs) of mackerel treated with high hydrostatic pressure (HHP) during storage at 4°C.

Storage period (days)	Treatment (bar)	VOCs (Area × 10^5^)			

		Ethanol	2-Butanone	3-Methylbutanal	Trans-2-pentenal
0	Control	315.16±110.78	33.76±2.34	ND^1)^	0.65±0.36
	2000	195.61±56.46	31.78±1.43	ND	0.21±0.09
	3000	286.99±26.97	42.58±13.26	ND	0.48±0.01
	4000	326.32±27.30	30.51±1.30	ND	0.32±0.07
10	Control	1874.88±166.37	41.27±12.93	252.27±20.39	5.83±2.03
	2000	183.65±31.01	60.86±10.98	26.91±3.71	4.52±1.09
	3000	296.78±34.88	85.00±4.78	39.20±4.72	7.80±1.45
	4000	309.74±31.41	65.99±3.46	28.03±2.76	4.84±0.54
20	Control	2455.97±129.70	104.38±6.58	391.35±39.54	21.53±4.06
	2000	365.71±19.27	75.62±6.48	45.62±10.24	5.73±0.001
	3000	438.10±10.39	72.04±0.84	39.84±0.09	3.92±1.32
	4000	413.20±28.04	63.85±0.74	32.94±1.42	1.70±0.81

Each value is the mean of duplicate measurement of pooled sample.

^1)^ ND, not detected
